# A Quantitative Assessment to Compare the Dimensions of the Alveolar Ridge Width Using Different Techniques for Implant Placement

**DOI:** 10.7759/cureus.46611

**Published:** 2023-10-06

**Authors:** Anam Nagib Mulla, Kaushik Kumar Pandey, Abhishek Gaur, Abhishek Kumar Katiyar, Pratibha Yadav, Ashmita Ghosh

**Affiliations:** 1 Department of Prosthodontics, Career Post Graduate Institute of Dental Sciences and Hospital, Lucknow, IND; 2 Department of Prosthodontics, Saraswati Dental College, Lucknow, IND

**Keywords:** dental implants, cbct, bone width, implant planning, ridge mapping

## Abstract

Background: The success of any dental implant surgery depends on the correct diagnosis and treatment planning.

Purpose: The aim of this study was to compare the dimensions of the alveolar ridge width using different techniques for implant placement.

Materials and methods: The study involved 27 partially edentulous subjects aged 18-50, including males and females. In this study, the dimensions of the ridge were evaluated by ridge mapping on a cast, ridge mapping using a bone caliper, and ridge mapping with the help of an occlusal radiograph. All three methods were compared with ridge mapping by cone beam computed tomography (CBCT). For each subject, the site of implant placement was marked on the study model. Alveolar ridge measurement was done in the mouth by a bone caliper under local anesthesia with the help of a stent with a hole. Ridge mapping on a cast was done after sectioning the cast and marking with the help of a periodontal probe and stent. Ridge mapping was done on an occlusal radiograph by converting an acetate stent into a radiographic stent. Finally, CBCT was taken for each patient for ridge mapping. All four readings were tabulated.

Results: Comparing the mean alveolar ridge width of four groups, ANOVA showed significantly different alveolar ridge width among the groups (F=7.89, p<0.001). The validity (accuracy and precision) of ridge mapping on a cast, ridge mapping using a bone caliper, and occlusal radiograph against the CBCT (gold standard) was done using concordance correlation analysis. The concordance correlation analysis showed the highest association (ρ=0.8196) and precision (ϸ=82.61%) of ridge mapping using a bone caliper with CBCT. However, the accuracy of ridge mapping on a cast (Cb=99.42%) was the highest, followed by ridge mapping using a bone caliper (Cb=82.61%). The analysis concluded that both techniques are equivalent to CBCT and can be used interchangeably.

Conclusion: The mean alveolar ridge width of the occlusal radiograph was the highest, followed by CBCT, ridge mapping on a cast, and ridge mapping using a bone caliper the least (occlusal radiograph > CBCT > ridge mapping on cast >ridge mapping using bone caliper). But at the same time, it can also be used interchangeably.

## Introduction

In the past, humans have attempted to replace missing or diseased tissues with natural or synthetic substances like carved ivory, bone, and natural extracted teeth. An alternative attachment mechanism was an accidental finding by Prof. Per Ingvar Branemark and his colleagues during the 1950s and 1960s. Since then, this metallic structure today called ‘implants’ has become incorporated into the living bone in a way formerly believed to be impossible. Branemark called it 'osseointegration' [1].

Since then, dental implant therapy has been increasingly and frequently used for the rehabilitation of missing dentition, replacing conventional therapies in the area of complete and partial edentulism as well as for single-tooth anodontia [2]. Dental implants are gaining immense popularity and wide acceptance because they not only replace lost teeth but are also permanent restorations that do not interfere with oral function or speech or compromise the self-esteem of patients. It is also important to place the implants in the maxilla and the mandible with a high degree of precision [3]. However, placing a dental implant is not an isolated event but a result of cautious presurgical planning [4]. The assessment includes patients' histories, desires, and preferences; extra and intraoral examinations; study casts; and radiographs [5]. The purpose of imaging the implant site is to decide whether implant treatment is appropriate for the patient and to know the location of vital anatomical structures such as the inferior alveolar nerve and the extension of the maxillary sinus for the assessment of bone quantity such as the height of the alveolar process, the bucco-lingual width, angulation, and the detection of possible undercuts and concavities.

Again, we must identify any possible pathological conditions and estimate the length and width of the implant to be inserted. We should decide the appropriate number of implants, the location and orientation, and the possible need for additional treatment before implant placement, for instance, bone augmentation procedures, to estimate the prognosis [3].

There are various radiographic methods available, which range from the simplest (intraoral films) to the more complex utilization (computed tomography) [6]. Two-dimensional (2D) imaging modalities provide information about the length of the bone and visualization of nerves and vessels but do not provide information about the width of the bone at the implant site [2]. As the width of the alveolar bone is important for implant placement, radiographic as well as manual techniques can be used to evaluate the width of the ridge.

Clinical procedures like ridge mapping were registered by Wilson in 1989 and Taxler in 1992, who suggested this to be a reliable method to evaluate bone availability for dental implant surgery [4]. The measurement of the alveolar ridge dimension can be accomplished using ridge mapping calipers under local anesthesia [1]. Ridge mapping, along with panoramic and intraoral radiographs, could be adequate to measure sites showing little bone resorption and in sites where there is no risk of damage to anatomical structures like the maxillary sinus or inferior alveolar nerve [4].

Schwarz and other researchers introduced cone beam computerized tomography (CBCT) as an auxiliary test in pre-surgical planning for dental implant treatment. The role of CBCT in providing accurate linear measurements of height and width and three-dimensional (3D) evaluation of the alveolar ridge with the fabrication of surgical guides is also useful during the placement of implants [7].

Therefore, this study was planned for a comparative evaluation of different techniques for bone evaluation to find out the most suitable, simple, and accurate technique that can be used in most dental clinics for dental implant surgery.

## Materials and methods

This study was conducted in the Department of Prosthodontics, Career Post Graduate Institute of Dental Sciences and Hospital, Lucknow, India. The study involved 27 partially edentulous subjects aged 18-50, including males and females. Written consent was obtained from all the subjects after explaining the procedure in detail. Subjects included in this study were cooperative and of Indian origin and needed treatment with dental implants in Kennedy Class III situations. Immunocompromised patients, pregnant women, and patients with poor oral hygiene were excluded. A total of 35 partially edentulous sites were evaluated as present in either of the two arches. Ethical clearance was obtained from the ethical committee of the institution (approval no.: CPGIDSH/18).

Steps

Fabrication of the Prosthetic Stent

**Figure 1:**
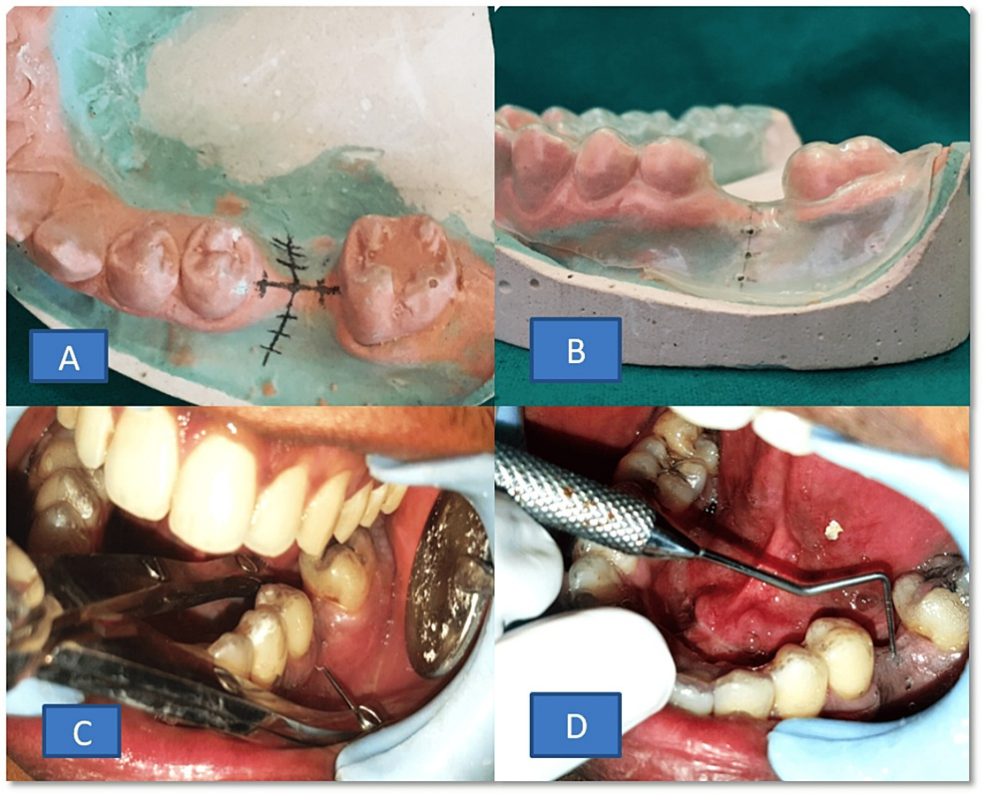
1A: marking of the implant site; 1B: making of the prosthetic stent and guided holes; 1C: alveolar ridge measurement by a bone caliper under local anesthesia; 1D: alveolar ridge mapping using a periodontal probe under local anesthesia

**Figure 2:**
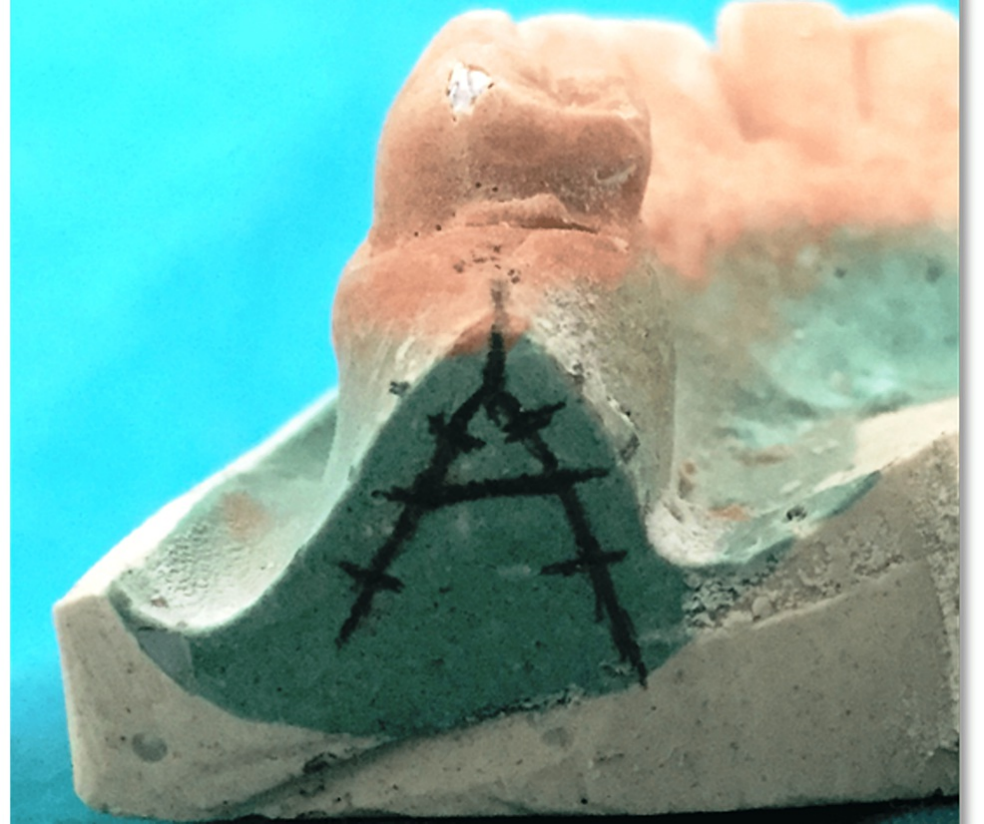
Transfer of the ridge map reading to the patients’ dental stone cast

Preliminary maxillary and mandibular diagnostic alginate impressions were made, and models were poured with dental stone. In the study models, a point (i.e., point 0) was marked on the crest of the ridge, marking the position where the implant had to be placed. Thereafter, three points were marked on both buccal/labial and lingual/palatal aspects at a distance of 3 mm from each other. Thus, points one, two, and three marked on both buccal/labial and lingual/palatal aspects were 3 mm, 6 mm, and 9 mm away from point 0, respectively. A line was drawn through these points to serve as a reference for the sectioning of the cast for the transfer of ridge map readings. A vacuum-formed stent was prepared on the study model for each patient, covering the edentulous ridges. Then, 1 mm-diameter guide holes were drilled for all the marked points through the stent using a micromotor straight fissure bur. Vacuum-formed acetate stents were disinfected using a 5% povidone-iodine solution. Patients were given infiltration local anesthesia and were then made to wear the vacuum stent. With the help of the bone caliper, readings will be taken through the stent orifices at point two. This point will be used for comparing all the readings. With the help of the periodontal probe in the guided holes of the vacuum stent, the thickness of the mucosa was measured at the crest of the ridge, point one, point two, and point three on both buccal and lingual aspects (i.e., a total of seven readings will be made). The readings were transferred to the dental stone cast forridge mapping. The same individual did the readings for each subject for the ridge mapping on the same day. The cast was sectioned using a dental saw on the line that was drawn across the study cast through the points, and the readings were transferred. A dotted line was joined to give the surface topography of the alveolar ridge. A horizontal line was drawn at point two to get the measure of the bone width (Figures 1, 2).

Alveolar Ridge Measurement by Intraoral Periapical Radiograph in Occlusal Projection

**Figure 3:**
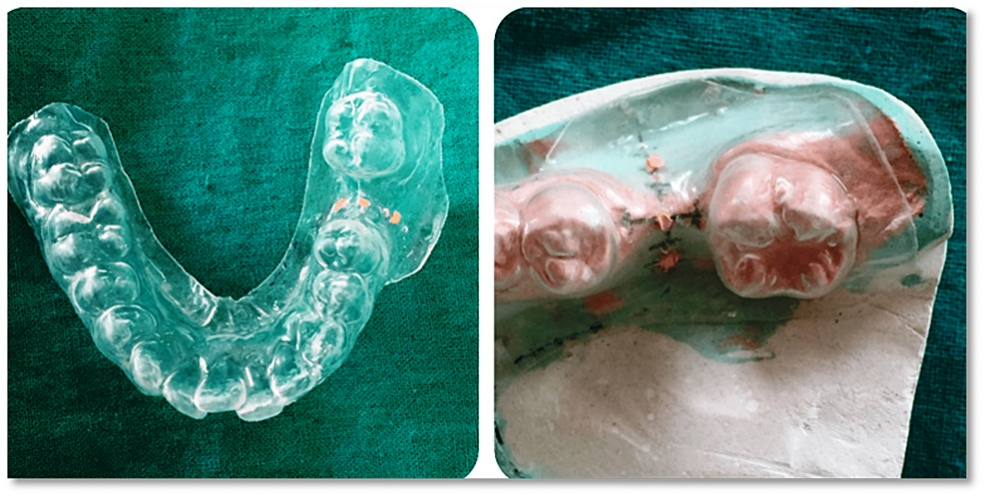
Modification of a prosthetic to a radiographic stent using gutta-percha

**Figure 4:**
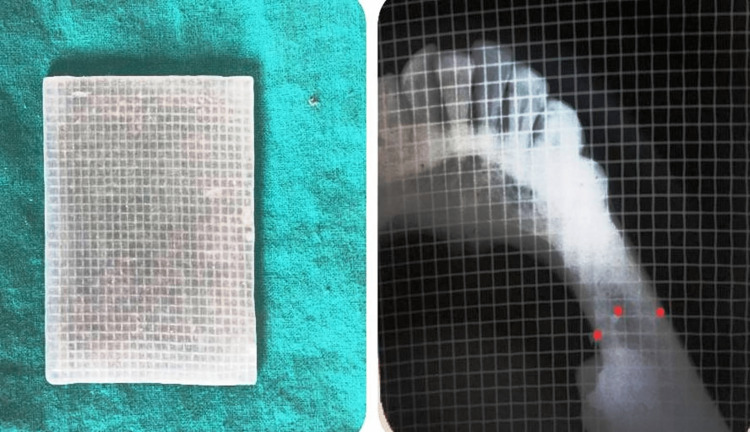
The image on the right shows a customized radiographic mesh, and the image on the left shows alveolar ridge measurement by intraoral periapical radiograph in the occlusal projection (red dots showing marking).

The vacuum-formed prosthetic stent was modified using gutta-percha to serve as radio-opaque markers at the crest of the ridge and at point two. Patients were made to wear this radiographic vacuum stent. A custom-made X-ray mesh gauge of the size of the occlusal radiograph was made (76 mm x 57 mm). The radiographic machine (KaVo FOCUS, KaVo Kerr Group, Charlotte, USA) was set at 75 kilovoltage (kVp) and 7 milliamperes (MA) for 0.8 seconds, and a radiograph was taken. The radiographs showed the radio opacity of the gutta-percha and the mesh markings. The occlusal marking showed the prosthetic center, and the mesh markings enabled us to calculate the width of the ridge at point two (Figures 3, 4).

Alveolar Ridge Measurement by CBCT

**Figure 5:**
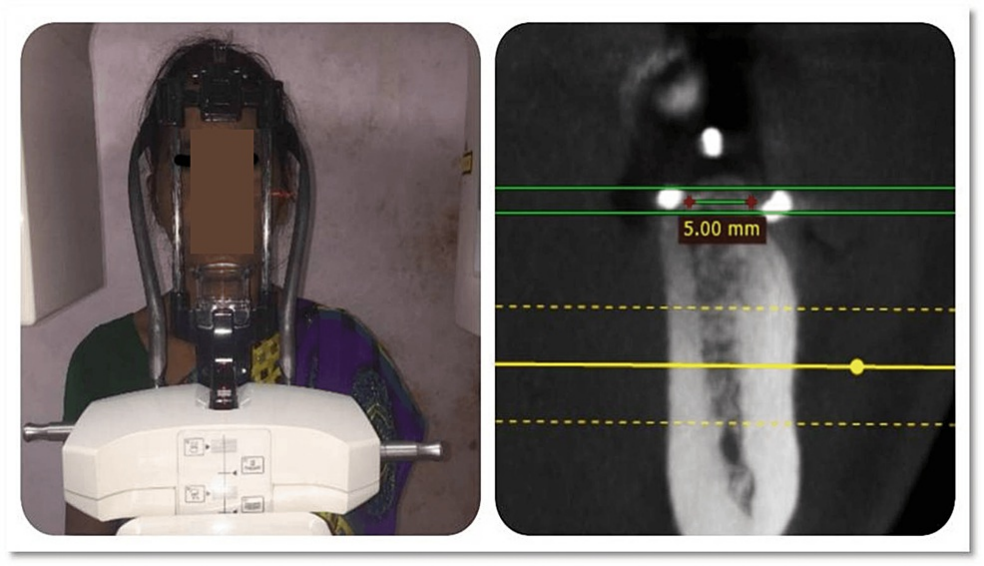
Alveolar ridge measurement by cone beam computerized tomography (CBCT)

The CBCT was done in the Department of Oral Medicine and Radiology at King George's Medical College, Lucknow. The patients were made to wear the radiographic vacuum stent and be exposed while standing with the Frankfurt-Horizontal plane parallel to the floor. The CBCT machine Carestream (Carestream Health, Gland, Switzerland) was set at 160-90 kVp and 2-15 MA for 12-28 seconds. Through the use of CBCT software RadiAnt (Medixant, Poznan, Poland), the alveolar ridge width at point two was measured. The readings in the software were taken by first drawing two parallel lines passing through the superior and inferior borders of the gutta-percha, and then a third line was drawn that was parallel and equidistant from them to give us the width of the ridge (Figure 5).

All the data were tabulated, and statistical analysis was done.

## Results

**Figure 6:**
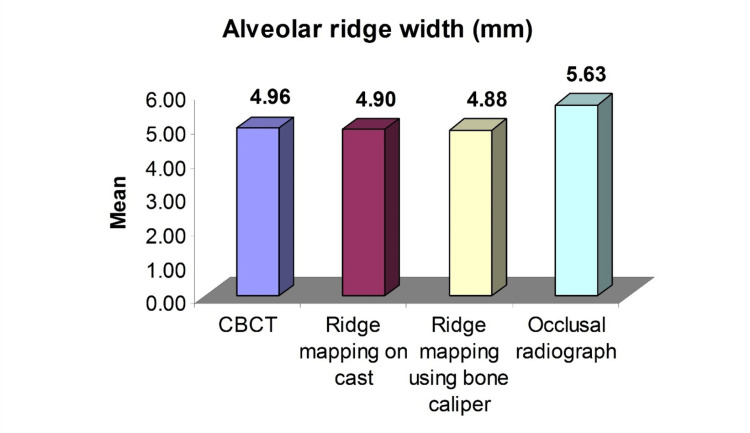
Mean alveolar ridge width of the four groups

Of the total 35 sites evaluated, 14 (40.0%) were located in the maxillary arch, and 21 (60.0%) were located in the mandibular arch. Among these, 30 (85.7%) were with adequate ridge height (h ≥ 9mm, i.e., good ridge) and five (14.3%) were with inadequate ridge height (h<= 5mm, i.e., poor ridge). An analysis was done of the ridge having adequate height (h ≥ 9mm). The quantitative analysis of the alveolar ridge width (mm) of the four groups was done using ANOVA. Tables 1-2 and Figure 6 show the comparative evaluation of the mean alveolar ridge width of four groups and show significantly different alveolar ridge widths among the groups (F=7.89, p<0.001).

**Table 1:** Alveolar ridge width of four groups and their comparison by ANOVA (F-value) CBCT: cone beam computed tomography

Groups	Alveolar ridge width (Mean ± SE, n=30)	F-value	P-value
CBCT	4.96 ± 0.13	7.89	<0.001
Ridge mapping on the cast	4.90 ± 0.12		
Ridge mapping using a bone caliper	4.88 ± 0.12		
Occlusal radiograph	5.63 ± 0.13		

**Table 2:** The validity of ridge mapping on a cast, ridge mapping using a bone caliper, and occlusal radiograph against CBCT (gold standard) using concordance correlation analysis CBCT: cone beam computed tomography systems

Statistics	CBCT vs.		
	Ridge mapping on a cast	Ridge mapping using a bone caliper	Occlusal radiograph
Sample size	30	30	30
Concordance correlation coefficient (ρ)	0.7647	0.8196	0.5301
95% CI of ρ	0.57 to 0.88	0.66 to 0.91	0.32 to 0.69
Pearson ϸ (precision)	0.7691	0.8261	0.7753
Bias correction factor Cb (accuracy)	0.9942	0.9921	0.6837

The mean alveolar ridge width of the occlusal radiograph was the highest, followed by CBCT, ridge mapping on a cast, and ridge mapping using a bone caliper the least. The validity (accuracy and precision) of the three methods against the CBCT (gold standard) was done using the concordance correlation analysis shown in Table 2.

The concordance correlation analysis showed the highest association (ρ=0.8196) and precision (ϸ=82.61%) of ridge mapping using a bone caliper with CBCT. However, the accuracy of ridge mapping on a cast (Cb=99.42%) was the highest, followed by ridge mapping using a bone caliper (Cb=82.61%). The analysis concluded that both techniques are equivalent to CBCT and can be used interchangeably (Tables 1-2, Figure 6).

## Discussion

In all phases of clinical dentistry, careful diagnosis and treatment planning result in a more predictable outcome [1]. Dental implants serve as the best choice in treatment for the replacement of missing teeth [8]. The fabrication of an implant-supported single tooth restoration, both aesthetically and functionally, depends on the ridge morphology and the orientation of the implant. The placement of dental implants requires meticulous planning and careful surgical procedures [1]. The success of a dental implant depends on the quantity and quality of the jaw bone and also on the precise radiographic visualization of anatomic structures and pathological conditions [9,10].

In pre-implant surgical design, the existing bone quality is determined by recording the width of the alveolar bone and its morphology [8]. This can be visualized using study models along with the diagnostic wax-up [1]. Most of the morphological views, such as bone undercuts, that are not directly visible in clinical examinations become visible with cross-sectional imaging. Thus, imaging potential recipient sites should provide accurate information for the precise placement of implants in the correct three-dimensional position [8]. There are various techniques that have been used clinically and radiographically in assessing the width of the alveolar ridge, like ridge mapping, trans-tomography, direct caliper measurement following surgical exposure, intra-oral periapical radiography in occlusal projection, orthopantomogram (OPG), CT scan, and finally CBCT.

Wilson and Taxler suggested ridge mapping as a reliable method to measure transversal alveolar bone [4]. Ridge mapping is a procedure that allows the surgeon to determine the thickness or width of the alveolar bone before the mucoperiosteal flap is reflected during surgery. This technique involves a series of measurements by a specially designed caliper. The sharp points of the caliper penetrate the anesthetized mucosa until the surface of the bone is reached. A millimeter scale near the handle end of the caliper will give an accurate reading of the ridge thickness. The ridge mapping method has the advantage of being simple to use and avoiding exposure to radiation for the patient [1]. However, it is to be kept in mind that ridge mapping may give erroneous readings, as suggested by Shirkar R. Desai [2].

A radiographic or surgical template plays a fundamental role in the ideal positioning and installation of implants for the use of prosthetic restoration. Radiographic stents are used for a more accurate assessment of the width and height of the available bone prior to treatment. After placing the stent intraorally, the patient undergoes radiography. By doing so, the radio-opaque markers are visualized on diagnostic radiographs planned for implant placement or surgical procedures [11]. The main drawback of all radiographs is the 2D imaging, unlike CBCT, which gives 3D visualization. Panoramic radiographs provide a good two-dimensional overview to facilitate the detection of pathologies in the jaw bone and the assessment of bone quantity in the vertical and mesiodistal dimensions. Panoramics are therefore considered a standard radiographic examination for the initial diagnosis and implant treatment planning [5].

In dentistry, periapical radiographs are routinely prescribed, as are OPGs, because periapical radiographs are unable to produce cross-sectional information. Occlusal radiographs are used to determine the faciolingual dimensions of the mandibular alveolar ridge [2]. Mandibular occlusal radiographs have an orthogonal projection, i.e., at right angles to the x-ray beam, and are less distorted [3,12]. Maxillary occlusal radiographs are inherently oblique, i.e., at an angle of 45° to the beam, and are of little use because of their distortion. These have little quantitative use in implant dentistry for either determining the geometry or degree of mineralization of the implant site [12]. However, the mandibular alveolus generally flares anteriorly and demonstrates lingual inclination posteriorly, producing a distorted image of the alveolus, which is of little use in implant dentistry [3].

While planning a dental implant, a three-dimensional orientation can be difficult to ascertain due to morphologic changes occurring due to edentulism and leading to varying degrees of resorption [5]. Tomograms before dental implant placement will help determine the height, width, inclination, and undercut of the alveolar bone, as well as the location of anatomical structures [2]. Computer-assisted implant planning on 3D models allows the optimal assessment and investigation for implant placement. The use of stereolithographic guides for the placement of dental implants is designed to provide greater control and eliminate the risks that are involved in standard implant surgery [2].

This relatively modern, state-of-the-art imaging technology has added another dimension to dental radiography and is quickly becoming the gold standard for radiographic imaging in dentistry. Maxillofacial CBCT imaging provides very accurate, submillimeter-resolution images of great diagnostic quality, enabling 3D visualization of the complex osseous structures of the maxillofacial region [13]. In this study, CBCT is taken as the gold standard for alveolar ridge mapping, as many researchers have proved it to be a standard modality for the measurement of available bone for implant planning. All three methods of ridge mapping were compared with the CBCT [14-22].

The quantitative analysis of the alveolar ridge width (mm) readings of the four groups was done. The highest mean (±SE) 5.63 ± 0.13 mm of the alveolar ridge width was of the occlusal radiograph, followed by CBCT (± SE) 4.96 ± 0.13 mm, ridge mapping on a cast (± SE) 4.90 ± 0.12 mm, and ridge mapping using a bone caliper (±SE) 4.88 ± 0.12 mm the least (occlusal radiograph > CBCT > ridge mapping on a cast >ridge mapping using a bone caliper). Comparing the mean alveolar ridge width of the four groups, ANOVA showed significantly different alveolar ridge width among the groups (F=7.89, p<0.001) (Table 1, Figure 6).

In this study, the results showed that the mean alveolar ridge width of the ridge mapping on a cast was the closest and the occlusal radiograph was the farthest when compared to CBCT gold standard observations. So the most reliable method after CBCT is ridge mapping on a cast, followed by ridge mapping by a bone caliper. Occlusal radiography is the least reliable method [23].

The quantitative analysis was also done using concordance co-relation analysis to check the validity and accuracy between the groups. It showed the highest association (ρ=0.8196) and precision (ϸ=82.61%) of ridge mapping using a bone caliper with CBCT. However, the accuracy of ridge mapping on a cast (Cb=99.42%) was the highest, followed by ridge mapping using a bone caliper (Cb=82.61%). Thus, the analysis concluded that both techniques are equivalent to CBCT and can be used interchangeably (Table 2).

Chugh A et al. and Mootha A et al. did a study using direct ridge mapping as a gold standard in which they concluded that ridge mapping done by transferring the readings on the sectioned cast had no significant difference from the gold standard [1, 2]. Castro-Ruiz et al. compared CBCT with ridge mapping using a caliper for estimating the alveolar ridge width and concluded that there was no statistically significant difference between the readings [4]. Eachempati P et al. concluded that there was a moderate correlation between ridge mapping with the cast sectioning method and the CBCT as the gold standard value. The mean difference found was about 1.2mm [22]. Dave B et al. found that there was no statistically significant difference (p >0.05) in the readings obtained between CBCT, ridge mapping technique, and direct surgical exposure technique [14].

## Conclusions

The width of the ridge evaluated by ridge mapping on a cast was the closest, followed by ridge mapping using a bone caliper, and then an occlusal radiograph against the CBCT (gold standard). The highest association and precision of ridge mapping using a bone caliper were seen with CBCT. However, the accuracy of ridge mapping on a cast was the highest, followed by ridge mapping using a bone caliper. Thus, in this study, it was concluded that both techniques are equivalent to CBCT and can be used interchangeably.

A CBCT could definitely be used on a regular basis for a single implant or multiple implants. Because it is a 3D measurement of the bone, it is suggested to be used in cases of the presence of ridge concavities or irregularities, in cases where augmentation is required, proximity to vital structures like the maxillary sinus and inferior alveolar nerve canal is avoided, and where the vestibular depth is inadequate and bone mapping cannot be performed.
